# Seroprevalence of leptospirosis in an endemic mixed urban and semi-urban setting—A community-based study in the district of Colombo, Sri Lanka

**DOI:** 10.1371/journal.pntd.0008309

**Published:** 2020-05-19

**Authors:** Senaka Rajapakse, Praveen N. Weeratunga, Krishan Balaji, Kyra Charmaine Ramchandani, Udani Savbhagya de Silva, Shenali Avishka Ranasinghe, Dinesh Gunarathne, Pasindu P. B. Wijerathne, Narmada Fernando, Shiroma M. Handunnetti, Sumadhya Deepika Fernando

**Affiliations:** 1 Department of Clinical Medicine, Faculty of Medicine, University of Colombo, Colombo, Sri Lanka; 2 Institute of Biochemistry, Molecular Biology and Biotechnology, University of Colombo, Colombo, Sri Lanka; 3 Smallholder Tea and Rubber Revitalization (STARR) Project, Ministry of Plantation Industries, Colombo, Sri Lanka; 4 Department of Parasitology, Faculty of Medicine, University of Colombo, Colombo, Sri Lanka; University of Connecticut Health Center, UNITED STATES

## Abstract

Leptospirosis is endemic in Sri Lanka. There is a need for updated seroprevalence studies in endemic areas, to improve the understanding of disease dynamics, risk factors, control methods, and for clinical diagnosis. The cut-off titres for the microscopic agglutination test (MAT) for diagnosis of acute leptospirosis depend on community seroprevalence, and can vary based on locality and serovar. This study aimed to identify the seroprevalence, geographical determinants, and associations of seropositivity of leptospirosis in the district of Colombo in Sri Lanka, and to determine diagnostic cut-off titres for MAT in the community studied. This study utilized a stratified cluster sampling model in the Colombo district of Sri Lanka, to sample individuals living in urban and semi-urban areas. Serovar specific MAT titres were measured on recruited individuals using a panel of saprophytic (*Leptospira biflexa)* and 11 pathogenic *Leptospira* spp. serovars. Associations between environmental risk factors and MAT positivity were examined, with location mapping using GIS software. A total of 810 individuals were included. The mean age was 51.71 years (SD 14.02) with male predominance (60%). A total of 429 (53%) tested positive at a titer of 1/40 or more for the saprophytic *Leptospira biflexa* serovar Patoc. Pathogenic serovar MAT was positive at a titer of 1/40 or more for at least one serovar in 269 (33.2%) individuals. From the perspective of screening for clinical disease, serovar-specific cut-off titres of 1/80 for *Leptospira* spp. serovars Hebdomadis, Icterohaemorrhagiae, Pomona, Ratnapura and Patoc, 1/160 for serovars Pyrogenes and Cynopteri, and 1/40 for other serovars were determined, based on the 75^th^ quartile MAT titre for each serovar. Serovar Pyrogenes (15.9%) had the highest seroprevalence, with serovars Ratnapura, Bankinang and Australis accounting for 9.9%, 9.6% and 9.3% respectively. When the proposed new cut-offs were applied, Bankinang(9.6%) Australis(9.3%), Pyrogenes(6.9%) and Ratnapura(6.9%) were the most prevalent serovars. No significant differences in seroprevalence or serovar patterns were noted between urban and semi-urban settings. Individuals seropositive for Australis, Ratnapura and Icterohaemorrhagiae were clustered around main water bodies as well as around smaller tributaries and paddy fields. Those positive for the serovar Pyrogenes were clustered around inland tributaries, smaller water sources and paddy fields. Associations of MAT positivity included high risk occupational exposure, environmental exposure including exposure to floods, bathing in rivers and lakes, using well-water for bathing, contact with stagnant water, propensity to skin injuries, presence of rats in the vicinity, and proximity to water sources. For pathogenic serovars, high-risk occupational exposure remained statistically significant following adjustment for other factors (adjusted OR = 2.408, CI 1.711 to 3.388; p<0.0001; Nagelkerke R^2^ = 0.546). High risk occupational exposure was determined to be independently associated with seropositivity. Baseline community MAT titres vary according to serovar, and presumably the locality. Testing against saprophytic serovars is unreliable. Thus, diagnostic MAT titre cut-offs should be determined based on region and serovar, and the use of a single diagnostic MAT cut-off for all populations is likely to result in false negatives.

## Author summary

Leptospirosis is a bacterial infection found widely among animals, which can also infect humans. Human disease results in fever, with acute damage to the liver and kidney occurring in severe cases. Around 58,900 deaths are estimated to occur worldwide from leptospirosis. In Sri Lanka, where the disease is widely prevalent, leptospirosis is commonly seen as a disease of farmers. Humans are infected through contact with rat urine, especially during paddy cultivation. In recent times, increasing numbers of cases are being reported in urban and semi-urban areas, as overcrowding, flooding, and poor sanitation provide an environment conducive to the transmission of leptospirosis. There are many serological types of leptospirosis, and the prevalence of these different serological variations (serovars) differ according to locality and animal host. The disease manifestations vary depending on, among other factors, the infecting serovar. Leptospirosis is commonly diagnosed using the microscopic agglutination test (MAT), which tests for both IgM and IgG antibodies. There is little information available about the baseline presence of antibodies to leptospirosis in community settings. In this study, a representative sample from the community was studied to determine the seroprevalence of leptospirosis (presence of antibodies to leptospirosis), from the district of Colombo in Sri Lanka. The Colombo district represents an urban/semi-urban area of Sri Lanka, where overcrowding is common, and flooding takes place often. Leptospirosis has shown a sharp increase in the Colombo district in recent years. The aim of this study was to determine two important aspects; the patterns of seroprevalence of common serological types, relating them to geographical and other risk factors, and to determine baseline cut-off values for antibody levels in the community. The latter is of great importance when using MAT to diagnose patients presenting with acute disease. This study found a relationship between the presence of seroconversion to pathogenic *Leptospira* types and high risk occupational exposures, environmental exposures including exposure to floods, bathing in rivers and lakes, using well water for bathing, contact with stagnant water, propensity to skin injuries, presence of rats in the vicinity, and proximity to water sources. It was also found that seropositivity for pathogenic *Leptospira* was lower than that for non-pathogenic species, reinforcing the importance of performing serovar-specific MAT for diagnosis. The cut-off titres for different serovars were found to differ, and were lower than the titres of ≥1/400 or ≥1/320 which are currently recommended by the World Health Organization and by local reference laboratories, respectively. This finding has important implications when determining the MAT titre cut-off for confirming the diagnosis of acute leptospirosis in patients with a suggestive clinical presentation.

## Introduction

Leptospirosis is a zoonotic disease caused by spirochaetes of the genus *Leptospira*, and is a disease of worldwide importance. Most mammals are hosts to the disease, and rodents are the main source of human infection. Transmission to humans occurs via contact with an infected animal, or through indirect contact via soil or water contaminated with urine from an infected animal. The disease has a wide spectrum of clinical manifestations, ranging from mild febrile illness to severe disease with multi-organ dysfunction. The global mortality and morbidity impact of the disease is estimated at 58,900 deaths per year, and 2.9 million disability adjusted life years [1, 2]. The estimated annual incidence of leptospirosis in Sri Lanka is 300 (95% CI 96·54–604.23) per 100,000 people [3]. In a study conducted during a period of flood outbreak, the incidence of leptospirosis requiring hospital admission was 52 (95% CI 51.69–52.57) per 100,000 people [4].

*Leptospira* can be classified according to genotypes and serovars. There are 64 species based on genotypic classification. A recent phylogenetic study proposed the classification of these 64 species into four subclades, i.e., P1, P2, S1 and S2, replacing the previously defined clusters of pathogenic (P1), intermediate (P2) and saphrophytic (S1 and S2) strains[5]. According to this classification, the P1 subclade is comprised of 17 species, P2 is comprised of 21 species, with the remaining 26 species belonging to S1 and S2 subclades.

Leptospires are long motile bacteria, which have a double membrane structure, comprised of an outer membrane which envelops the cytoplasmic membrane, and a peptidoglycan cell wall. The outer membrane is composed of lipopolysaccharides(LPS)[6, 7]. These LPS are the primary antigens, and show wide variability. Serological classification of *Leptospira* is based on their LPS structure and reactivity of antibodies to these varied antigens. Two strains with heterogeneity of 10% or more are considered to belong to different serovars, and a serogroup includes serovars with overlapping antigens. There are more than 300 serovars identified, and these are grouped into several serogroups; only a few of these are pathogenic[8]. The serovars commonly tested for in Sri Lanka, at the time of this study, were *Leptospira spp* serovars Australis, Bankinang, Bataviae, Canicola, Ratnapura, Hardjo, Icterohaemorrhagiae, Pyrogenes, Pomona, Hebdomadis and Cynopteri. The non-pathogenic serovar was *L*.*biflexa* serovar Patoc.

*Leptospira* infection shows natural nidality. Certain serovars are characteristically associated with different animal hosts, e.g., Hardjo, Hebdomadis, Sejroe, Pyrogenes, Autumnalis, Australis, Javanica, and Tarassovi are associated with cattle, Pomona and Australis with pigs and sheep, Tarassovi with pigs (in addition to cattle), and icterohaemorrhaghiae, Canicola and Grippotyphosa with all three farm animals[9–11]. Nearly every serovar has been found in rats, although geographical distributions vary, and Icterohaemorrhagiae is the most common in Sri Lanka[12]. A detailed review of these associations is beyond the scope of this paper. The importance of knowing this host-specific variability is that the identification of the infecting serovar may provide information about the possible local animal host.

Because of the diversity of *Leptospira*, the management and control of the disease is challenging, as these diverse serovars have unique reservoirs, ecological transmission, microbiological characteristics, and clinical and prognostic associations. The problems in curtailing the impact of the disease in different settings are probably related, at least to some extent, to poor understanding of the characteristics and seroprevalence of these serovars in the community setting. This is further complicated by the variety of reservoir animals acting as hosts to different serovars, and a range of different ecological systems that facilitate disease transmission in the animal–human interface.

There are few studies on the seroprevalence and associations of leptospirosis in Sri Lanka. Most community-based studies are from 1960–1970, and others are mostly based on hospital and patient data, which may not necessarily represent the true community characteristics of the disease. Traditionally, leptospirosis is a disease of paddy farmers in Sri Lanka. The Colombo district of Sri Lanka covers a land area of 676 km^2^, and has the highest population density in the country, i.e., 3438 persons per km^2^. The population of the Colombo district, according to the 2012 Sri Lanka Census of Population and Housing, was 2,324,349. Located in the low-country wet-zone of the island, the Colombo district bears the brunt of the South-West monsoonal rains, and in recent years has been one of the worst flood-affected districts. Being a typical urban/semi-urban area, with a dense population, poor water and sanitation services in some areas, and frequent flooding, it provides an environment conducive to the transmission of leptospirosis. Over the years, large numbers of patients have been reported from the Colombo District, and in 2010, the highest proportion of reported cases in the country were from this district (610 out of 4545, 13%).

While the microscopic agglutination test (MAT) is widely recognized as the reference standard for the diagnosis of leptospirosis, the cut-off titre considered for diagnosis of acute leptospirosis varies widely in different studies, and the World Health Organization defines this as 1/400[13]. In previous studies, however, varying cut-off titres have been used to determine seroprevalence ranging, from 1/40 to 1/100[14–19]. None of these cut-offs are based on baseline seroprevalence data, since the baseline cut-off titre for MAT has not been determined in a large community cohort; thus these cut-offs have, so far, been arbitrary. The baseline cut-off MAT titre in the community is also likely to vary according geographical location, as it is related to many factors such as animal reservoirs, serovar distribution, occupation, proximity to water sources, and many other uncharted determinants. MAT tests for both IgM and IgG antibodies, making it less adept at differentiating acute from previous illness. Knowing the cut-off point for MAT titres is of vital importance when using MAT to differentiate acute from prior illness, and this depends on the baseline seroprevalance of the disease in the community to which the patient belongs. In the past, MAT was mostly based on the saprophytic *L*.*biflexa*, however now serovar specific MAT panels are available. The use of a single standard MAT titre based on either pathogenic strains or pooled serovars for all patients is inappropriate. The authors postulate that the cut-off points for MAT titres vary from locality to locality, and from serovar to serovar, based on seroprevalence patterns in the community. Identifying this MAT threshold for individual serovars and for different localities is essential in order to differentiate acute infection from seroconversion due to prior infection, using MAT.

It is on this background that this study was positioned, aimed at identifying the baseline community seroprevalence of leptospirosis based on MAT, together with determining epidemiological and environmental risk factors for the disease in different localities and their relationships to prevalent serovars, in an urban/semi-urban setting. This information will potentially guide the implementation of targeted health interventions to reduce the occurrence of leptospirosis, create a more robust understanding of the disease, and also help interpret diagnostic MAT cut-off titres relevant to the locality. Furthermore, the methodology and findings will be instrumental in guiding further investigations in this area, both in Sri Lanka as well as in the global context.

## Methods

### Ethics statement

Ethics approval was obtained from the Ethics Review Committee of the Faculty of Medicine, University of Colombo (registration number ERC/14-010). Informed written consent was obtained from all participants.

### Characteristics of the study area

This descriptive cross-sectional study was carried out in the district of Colombo, situated in the Western Province of Sri Lanka, from 2015–2017. The district of Colombo comprises 13 Divisional Secretarial Divisions which are further subdivided into 566 ‘Grama Niladari’ (GN) Divisions (minor administrative divisions). The district is, for community healthcare administration purposes, divided into 19 ‘Medical Officer of Health’ (MOH) areas. MOH areas are administrative units below the district level, with a median population of about 50,000 and an average area of 208 km^2^. A MOH area is further subdivided into ‘Public Health Inspector’ (PHI) areas. The Colombo district is divided into 87 PHI areas. The GN division was used as the primary sampling unit for the purpose of the study.

### Sampling technique

Sample size was calculated according to the population formula $n=\frac{Z^{2}P(1-P)}{d^{2}}$, where n = sample size, Z = Z-statistic for a level of confidence, P = expected prevalence or proportion, and d = precision. The calculation was based on a maximum expected community seroprevalence reflected by leptospiral MAT positivity of 50% in order to obtain the maximum sample size. A confidence level of 95%, and a margin of error (precision) of 5% was used in the calculation. A two-stage cluster sampling method was used, taking into consideration a design effect of 2, and a 5% non-response rate. The estimated sample size based on the above calculation was 807 individuals.

### Data collection methods

The primary sampling cluster identified was a GN division (population approximately 3000), which is the lowest administrative area. Out of 566 GN divisions, 27 were selected randomly, using probability proportion to size (PPS) to compensate for differences in cluster sizes. From each cluster, 30 individuals were selected using simple random sampling. Lists of adult householders were obtained, and one member from each household was selected randomly, until 30 participants were recruited. If the selected householder was not present, data was collected on subsequent date by appointment. Individuals were initially screened for symptoms of leptospirosis within the past two weeks, and were excluded if any were present. Participants were informed about all aspects of the study, and written consent was obtained from those willing to participate. Anonymity of the participants and confidentiality of the data obtained were maintained. Demographic data, and information on exposure history, previous illness, current symptoms were collected from participants.

### Microscopic agglutination test (MAT)

The microscopic agglutination test (MAT) is a widely used reference test for antibody detection in leptospirosis. MAT was carried out at the National Reference Laboratory for Leptospirosis, Medical Research Institute, Colombo, Sri Lanka, using a panel of saprophytic and 11 pathogenic *Leptospira* reference serovars. MAT is a test method used to determine the circulating functional antibodies targeting the infecting serovar. The method utilizes the agglutinating technique, in which live leptospires are mixed with serial dilutions of serum collected from patients. Anti-leptospiral antibodies present in the serum cause leptospires to agglutinate, which is observed using dark field microscopy. Both circulating IgG and IgM antibodies are detected by this method. The result and titer are evaluated based on the degree of agglutination using dark field microscopy. The end-point is defined as dilution factor of serum that gives 50% of agglutination and 50% of free cells when compared to the control. Baseline positive MAT was defined as those with a MAT titre ≥1/40 to one or more serovars, and exact MAT titre values for each serovar were also measured. The starting titre was 1/20. Serial titres were not measured, as all samples were from asymptomatic individuals with no clinical features of leptospirosis within a 2-week time period. The pathogenic serovars included in the panel were pooled *L*. *spp* serovars Australis, Bankinang, Bataviae, Canicola, Ratnapura, Hardjo, Icterohaemorrhagiae, Pyrogenes, Pomona, Hebdomadis and Cynopteri. The non-pathogenic serovar was *L*.*biflexa* serovar Patoc.

### Geographical information systems (GIS) methodology

GPS (global positioning system) locations of the households of individuals where blood sampling was performed was obtained by site visits. Handheld GPS Revisers (Garmin 610) were used to obtain GPS locations. Using satellite images, water areas, paddy lands, wetlands and other land use patterns were digitized with the assistance of QGIS(qgis.org) and ArcGIS 10.5 software by Esri. For standardization, this digitization was corroborated with Government Survey Department digital land use data. Based on the GPS database and digitized land use data, buffer zones for 100m, 250m and 500m were created using ArcGIS 10.5 software proximity tools. Using buffers, maps showing individuals with MAT positivity were created in relation to water sources and possible transmission reservoir sites, and also to identify local MAT titre cut-offs, for pathogenic and non-pathogenic serovars, in the community studied.

### Statistical analysis

The association between outcomes and exposure variables were analyzed in two steps. Initial analysis included bi-variable comparison of individual exposure variables with outcomes by chi-square tests or logistic regression, followed by multivariable logistic regression. A manual forward and backward selection method was used to evaluate the association between exposure and confounding variables with the outcome. Exposure variables were entered in the model if the bivariable p-value was ≤0.2 or if they represented biologically plausible risk or confounding factors for the outcome, and were kept in the model if the likelihood ratio test was statistically significant at p≤0.05. The median and interquartile range was calculated for MAT titers, since the titre values are non-parametric, with cutoffs values for MAT titres determined based on values above the 75^th^ quartile.

## Results

### Basic demographic and clinical data

A total of 810 individuals were included in the study. The mean age of the sample was 51 years (SD 14), age range 18–89 years, and 60% were men. The majority were of Sinhalese ethnicity (693/810), which is consistent with the socio-demographic characteristics of the region selected for the study (Table 1). None of the patients had any symptoms which could be related to leptospirosis at the time of sample collection, or within a two-week period prior to and afterwards.

**Table 1:** Basic sociodemographic characteristics.

Characteristic		Mean (SD)
Mean age of the population (years)		51.71 (SD = 14.02)
Gender	Male	486 (60%)
	Female	324 (40%)
Ethnic group	Sinhala	693 (85.6%)
	Tamil	61 (7.5%)
	Muslim	56 (6.9%)
Population	Rural	298 (36.8%)
	Urban	512 (63.2%)

### Serological data based on a MAT cut-off of 1/40

**Fig 1:**
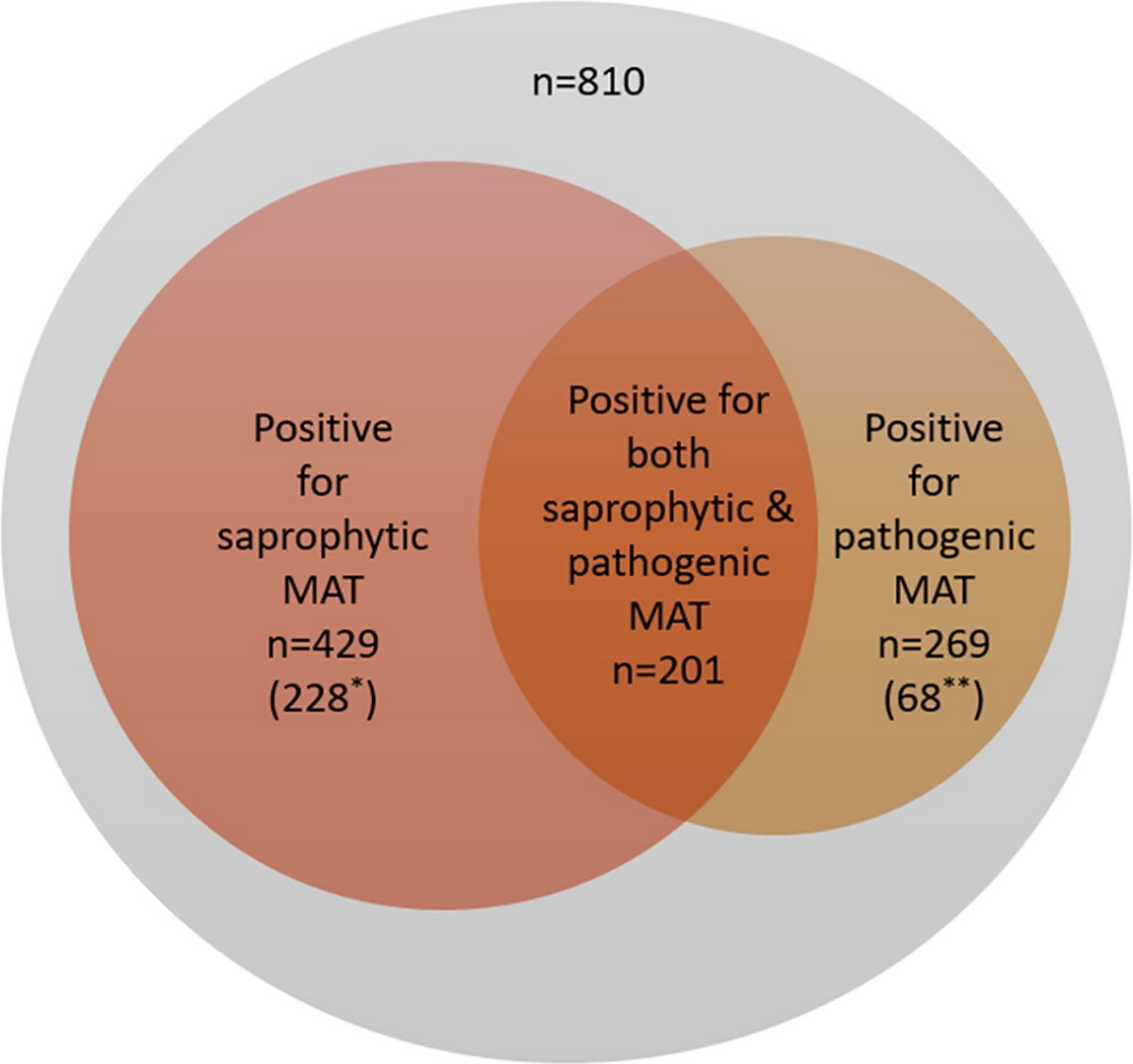
Venn diagram showing MAT positivity based on pathogenic serovar MAT and saprophytic serovar MAT. (*number positive for saprophytic serovar MAT only, **number positive for pathogenic serovar MAT only).

A total of 429 (53%) tested positive at a titer of 1/40 or more for the non-pathogenic *L*. *biflexa* serovar Patoc. Pathogenic serovar MAT were positive at a titer of 1/40 or more in 269 (33.2%) individuals. The highest documented titer was 1/1280. Most individuals had titers of 1/40 (43.8% or 1/80 (26.1%). Of the 269 individuals who were positive for pathogenic serovar MAT, 68(25.23%) were negative for saprophytic serovars. Similarly, 228 who were positive for saprophytic serovar MAT were negative for pathogenic serovar MAT. A total of 497 were positive with either saphrophytic serovar MAT, pathogenic serovar MAT or both (Fig 1).

The panel of pathogenic serovars included in MAT testing and observed positives are presented in Table 2. The distribution of titer values and their frequencies are also shown. The commonest was the serovar Pyrogenes (15.9%), with Ratnapura, Bankinang and Australis accounting for 9.9%, 9.6% and 9.3% respectively. The least common serovar was Hardjo (1.1%). Pyrogenes was the predominant serovar in 12/27 regional clusters, Australis in 4/27, Rathnapura in 5/27 and Bankinang in 7/27 clusters. Around half the individuals were positive for more than one pathogenic serovar (135/269, 50.1%).

**Table 2:** Serovar distribution and observed titers in the study population. Numbers under individual titres- number positive for titer/total number positive for that serovar, as percentage.

Serovar	Frequency (n)	Percentage out of total (n = 810)	1/40 (%)	1/80 (%)	1/160 (%)	1/320 (%)	1/640 (%)	Higher than 1/640 (%)
Australis	75	9.3	38.7	26.7	17.3	12.0	5.3	
Bankinang	78	9.6	47.4	26.9	15.4	8.1	5.1	
Bataviae	16	2.0	37.5	12.5	31.3	18.8	0.0	
Canicola	24	3.0	70.8	20.8	8.3			
Ratnapura	80	9.9	30.0	37.5	18.8	7.5	6.3	
Hardjo	9	1.1	100.0					
Icterohaemorrhagiae	45	5.6	31.1	37.8	20.0	0	11.1	
Pyrogenes	129	15.9	26.4	30.2	20.2	10.9	12.4	
Pomona	46	5.7	56.5	26.1	8.7	8.7	0.0	
Hebdomadis	71	8.8	38.0	33.8	7.0	8.5	9.9	2.8
Cynopteri	44	5.4	45.5	15.9	2.5	2.3		11.3

### MAT positivity based on population density

In areas of high population density, 138/410 (33.7%) individuals were positive for pathogenic serovar MAT and 208/410 (50.7%) for saprophytic serovar MAT. In low/intermediate population density areas 151/400 were positive for pathogenic serovar MAT (37.8%) and 244/400 were positive for saprophytic serovar MAT (61.0%). In both areas the most common pathogenic serovar was Pyrogenes.

### MAT titre cut-offs for individual serovars

The seroprevalence data of this study population represents baseline levels of MAT positivity in healthy individuals. Wide discrepancies are seen between cut-off titres used in seroepidemiological studies and those used for clinical diagnosis. The conventional cut-off MAT titre for diagnosis of leptospirosis in patients with a suggestive clinical picture varies in different studies, but ≥1/320 or ≥1/400 is considered the usual threshold, while epidemiological studies use a much lower titre threshold, i.e., 1/40–1/100.

**Fig 2:**
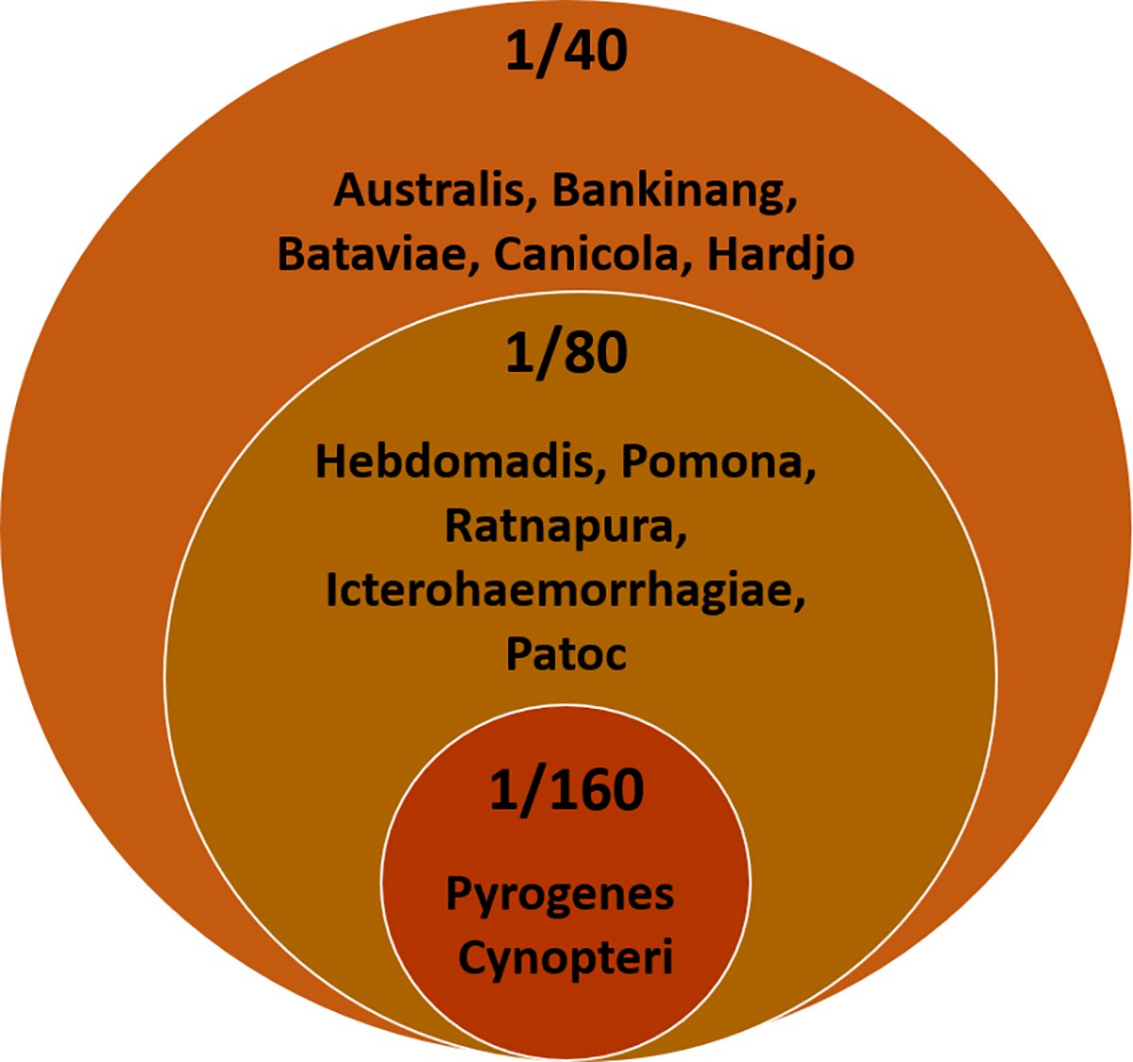
Suggested diagnostic cut-off titres for different serovars.

In this study, the 75^th^ quartile titers were selected as the cutoff for background seroprevalence for each serovar. These titres are postulated to represent cutoffs for the diagnosis of disease in clinical practice, in the community studied. Based on data presented in Table 3, a cut-off value of 1/80 was determined for serovars Hebdomadis, Icteroheamorrhagiae, Pomona, Ratnapura and Patoc while a value of 1/160 represented cutoffs for serovars Pyrogenes and Cynopteri. Diagnostic cutoff thresholds were recorded at 1/40 for all other serovars (Fig 2). When the proposed new cut-offs are applied to the cohort, the prevalence of the different serovars changes considerably, with Bankinang and Australis becoming the most prevalent serovars, and Pyrogenes moving down to third place (Table 4). This shows proof of concept that using appropriate cut-off can significantly change the regional prevalence of the disease.

**Table 3:** Analysis of MAT titers.

Serovar	Mean log	Log SD	Geometric mean (+/-2SD)	Median (+/-2SD)	25^th^ Q	50^th^ Q	75^th^ Q
Patoc (n = 600)	1.7591	0.44240	57.42 (+/-2.769*2)	40.00	20.00	40.00	80.00
Australis (n = 166)	1.5984	0.41127	39.66 (+/-2.570*2)	20.00	20.00	19.99	40.00
Bankinang (n = 169)	1.5700	0.37311	37.15 (+/-2.360*2)	20.00	20.00	19.99	40.00
Bataviae (n = 50)	1.5238	0.38373	33.40 (+/-2.420*2)	20.00	20.00	19.99	40.00
Canicola (n = 43)	1.5321	0.25303	34.04 (+/-1.790*2)	40.00	20.00	40.00	40.00
Ratnapura (n = 111)	1.7838	0.42000	60.78 (+/-2.630*2)	80.00	20.00	80.00	80.00
Hardjo (n = 25)	1.4094	1.40400	25.66 (+/-1.404*2)	20.00	20.00	19.99	40.00
Icterohaemorrhagiae(n = 84)	1.6594	0.42943	45.64 (+/-2.680*2)	40.00	20.00	40.00	80.00
Pyrogenes (n = 155)	1.9553	0.45439	90.21 (+/-2.840*2)	80.00	40.00	80.00	160.00
Pomona (n = 78)	1.6021	0.33612	40.00 (+/-2.160*2)	40.00	20.00	40.00	80.00
Hebdomadis (n = 92)	1.8278	0.47780	67.26 (+/-3.000*2)	40.00	40.00	40.00	80.00
Cynopteri (n = 61)	1.8389	0.62451	69.00 (+/-4.212*2)	40.00	20.00	40.00	160.00

**Table 4:** Adjusted seroprevalence for pathogenic serovars based on proposed cut-off titers.

Serovar	Proposed cut-off titre	Number positive	Percentage
Bankinang	1/40	78	9.6%
Australis		75	9.3%
Bataviae		16	2.0%
Canicola		24	3.0%
Hardjo		9	1.1%
Ratnapura	1/80	56	6.9%
Pomona		20	2.5%
Icterohaemorrhagiae		31	3.8%
Hebdomadis		44	5.4%
Pyrogenes	1/160	56	6.9%
Cynopteri		17	2.0%

### GIS data and maps

**Fig 3:**
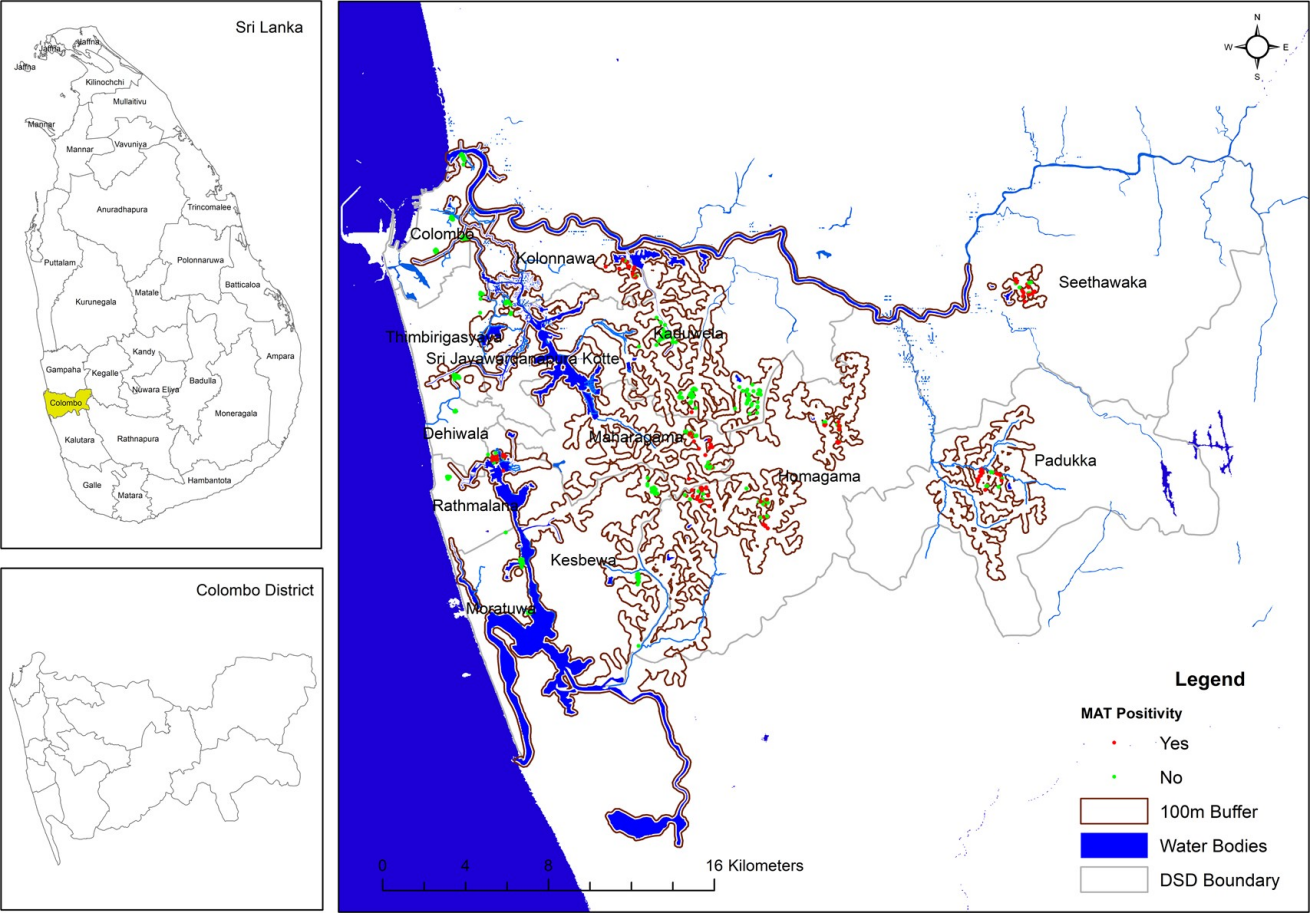
MAT positivity and distribution among water bodies. This image is licensed under the Creative Commons Attribution 4.0 International License (http://creativecommons.org/licenses/by/4.0/).

**Fig 4:**
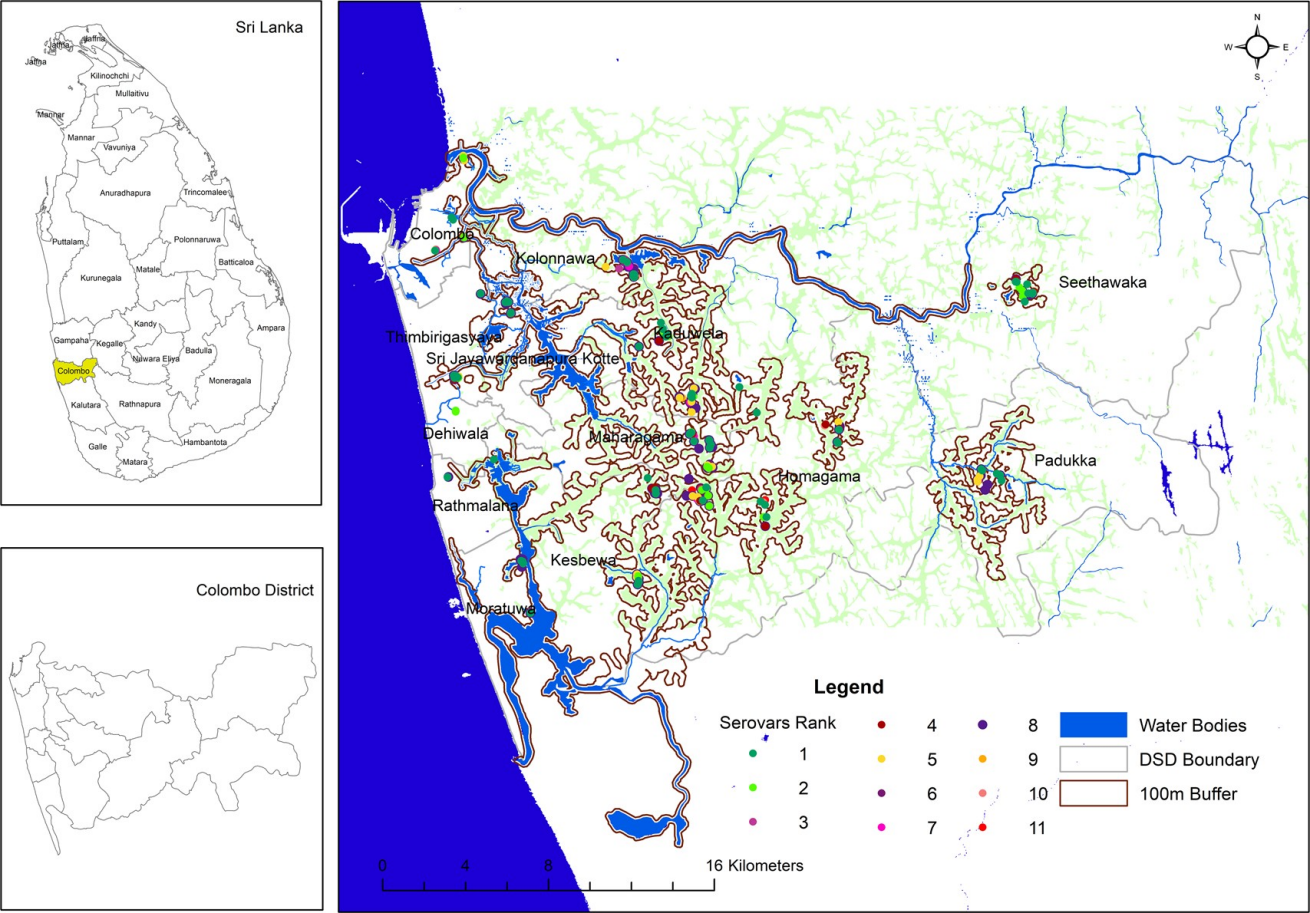
Pathogenic Serovar distribution among water bodies and paddy fields. Key to serovar numbers:1 –Australis, 2. Bankinang, 3 –Bataviae, 4 –Canicola, 5 –Ratnapura, 6—Hardjo, 7 –Icterohaemorrhagiae, 8 –Pyrogenes, 9 –Pomona, 10 –Hebdomadis, 11 –Cynopteri. Areas outlined in green indicates paddy fields and water tributaries. This image is licensed under the Creative Commons Attribution 4.0 International License (http://creativecommons.org/licenses/by/4.0/).

The GIS maps presented below (Figs 3 and 4) demonstrate clustering of MAT positives to water sources and paddy fields. Serovars Australis, Ratnapura and Icterohaemorrhagiae clustered around main water bodies such as rivers as well as inland, around smaller tributaries and paddy fields. Serovar Pyrogenes was noted to cluster around inland tributaries, smaller water sources and paddy fields.

### Non-pathogenic serovar positivity–univariate analysis

Univariate analysis was performed to evaluate the associations of socio-demographic data and risk exposures with positivity for the non-pathogenic *L*. *biflexa* serovar Patoc (Table 5). Individuals in the age groups 18–38 years, 39–65 years, and those engaging in high risk occupations (farming and other agricultural exposure, working in sugar cane plantations, garbage handlers, sewage workers and abattoir workers) had a statistically significant association with MAT positivity for the Patoc strain. No significant associations were noted for saprophytic MAT for individuals residing within buffer zones at 50m, 100m, 250m and 500m.

**Table 5:** Associations of non-pathogenic MAT positivity.

Association		MAT positive	P value
Age	18–38	57.3%	***0*.*034***
	39–65	60.2%	***0*.*002***
	> 65	34.6%	0.345
Gender	Male	42.7%	0.101
	Female	57.3%	
Income status	High	59.2%	0.402
	Low	53.6%	
Educational level	Pre-O/L	58.5%	0.463
	A/L and above	52.7%	
High risk occupations	Yes	62.7%	***0*.*002***
	No	37.3%	
Rats in the vicinity	Yes	52.6%	0.878
	No	53.2%	
Buffaloes in the vicinity	Yes	46.9%	0.481
	No	53.2%	
Exposure to floods	Yes	50.7%	0.537
	No	53.5%	
Contact with stagnant water	Yes	52.7%	0.930
	Yes	53.1%	
Garbage collections in proximity to house	Yes	60.4%	0.243
	No	51.8%	
Bathing and swimming in a river	Yes	1.30%	***0*.*046***
	No	98.7%	
Bathing from a well	Yes	48.9%	0.072
	No	57.4%	
Prone to skin injuries	Yes	55.0%	0.441
	No	52.1%	
Protective equipment use in risk occupations	Yes	57.3%	0.601
	No	52.2%	

### Seroprevalence data for high risk exposures

Stratification of the data for high risk exposures was performed. High risk exposures were defined as paddy farming, recreational activities in paddy fields/muddy grounds, contact with potentially contaminated water such as cleaning drains/wells, bathing and washing in small water streams, rivers and lakes, exposure to flood water, and contact with animals or animal tissues such as cattle and buffalo. Veterinarians, butchers, rodent control workers, and abattoir workers were considered to be at high risk. Pyrogenes was the most common serovar encountered in these risk groups and had seroprevalence ranging from 13.3% to 24.3%. Sampled individuals who frequently bathed using well-water had the highest seroprevalence rate. Individuals who reported the regular presence of buffaloes in the vicinity had a seroprevalence of 20.8% compared to those who reported rats in the vicinity (14.2%).

### Associations of pathogenic serovar positivity

Univariate analysis was performed for factors associated with pathogenic serovar positivity (Table 6). Male gender (p<0.001), low income status (p = 0.006), engaging in high risk occupations (p<0.001), bathing using well-water (p<0.001), swimming and bathing in local rivers and lakes (p = 0.041), contact with stagnant water (p < 0.001) and propensity to skin injuries (p <0.001) were associated with a positive pathogenic MAT. Interestingly, exposure to floods, and the presence of rats in the vicinity of households had negative associations with pathogenic serovar MAT. Associations of the four most common serovars are also presented in Table 6. GIS buffer zone analysis revealed significant associations for individuals residing within all zones <500m from water sources and paddy fields (p<0.05). Seroprevalence for pathogenic serovars was significantly higher within the buffer zones of 250m and 100m.

**Table 6:** Associations of pathogenic MAT positivity.

Association		MAT + (Pathogenic)	P value	Pyrogenes (p value)	Ratnapura (p value)	Australis (p value)	Bankinang (p value)
Age (years)	18–38	54.5%	0.215	0.142	0.235	0.425	0.812
	39–64	34.2%	0.421	0.254	0.124	0.574	0.124
	>65	31.5%	0.954	0.178	0.474	0.784	0.617
Gender	Male	44.8%	***<0*.*001***	***0*.*002***	***0*.*004***	***0*.*001***	***<0*.*001***
	Female	25.5%					
Low Income status	Yes	52.3%	***0*.*006***	0.178	***<0*.*0001***	0.968	0.688
	No	32.1%					
Educational level	No formal education	19.5%	0.056	0.267	0.267	0.910	0.606
	Formal education	33.9%					
High risk occupations	Yes	42.3%	***<0*.*001***	***<0*.*0001***	***<0*.*0001***	***0*.*001***	***<0*.*001***
	No	20.9%					
Rats in the vicinity	Yes	29.1%	***0*.*035***	0.239	0.711	0.066	0.950
	No	36.2%					
Buffaloes in the vicinity	Yes	43.8%	0.196	0.055	0.509	0.549	0.574
	No	32.8%					
Exposure to floods	Yes	25.0%	***0*.*019***	0.175	***0*.*009***	0.926	0.316
	No	35.0%					
Contact with stagnant water	Yes	47.3%	***<0*.*001***	***0*.*001***	***0*.*007***	***0*.*005***	***0*.*023***
	No	29.2%					
Garbage collections in proximity to house	Yes	39.6%	0.189	0.678	0.340	0.876	0.393
	No	32.3%					
Bathing and swimming in the river	Yes	80.0%	***0*.*041***	0.183	0.530	***<0*.*0001***	***0*.*023***
	No	35.9%					
Bathing from the well	Yes	46.0%	***<0*.*001***	***0*.*028***	0.548	0.286	0.166
	No	33.9%					
Prone to skin injuries	Yes	42.2%	***<0*.*001***	***0*.*006***	0.115	***0*.*022***	***0*.*023***
	No	29.2%					
Protective equipment use in risk occupations	Boots	33.3%	0.080	0.057	0.414	0.432	0.422
	Gloves	29.6%	0.524	0.752	0.245	0.582	0.254
	Other	48.9%	0.245	0.354	0.123	0.325	0.218
Non-use of chemoprophylaxis		55.6%	***<0*.*001***	***0*.*001***	***0*.*002***	***0*.*001***	***<0*.*0001***

### Regression analysis for independent associations of pathogenic MAT and non-pathogenic MAT

Logistic regression (LR) analysis with adjustment of risk factors for MAT positivity was performed. The independent variables included as covariates were high risk occupational exposures, environmental exposures (floods, bathing in rivers and lakes, using well-water for bathing, contact with stagnant water), propensity to skin injuries, presence of rats in the vicinity and GIS data on proximity to water sources. A backward LR methodology was utilized. High risk occupational exposures remained statistically significant following adjustment for other factors (adjusted OR = 2.408 CI 1.711 to 3.388; p<0.0001) an inverse relationship was noted with presence of rats in the vicinity. The Nagelkerke R squared statistic on the model was 0.546. In the non-pathogenic serovar analysis, high risk occupational exposures (adjusted OR = 1.553, CI 1.142 to 2.113; p = 0.023) and bathing with well water (adjusted OR = 1.566, CI 1.063 to 2.037) remained significant, after adjusting for other co-variates.

## Discussion

### Summary of findings

This study presents data on community seroprevalence of pathogenic and non-pathogenic serovars of leptospirosis, based on MAT, in the District of Colombo, Sri Lanka. Seroprevalence for the saprophytic serovar Patoc was noted at 53.0%, and for pathogenic serovars was 33.2%, with Pyrogenes emerging as the most prevalent (15.9%). Pyrogenes remained the most prevalent serovar in 12/27 clusters included, with other serovars such as Ratnapura and Australis emerging as the most prevalent in other clusters. Associations were generated for cases positive for non-pathogenic and pathogenic serovars with subsequent adjustment with logistic regression analysis. The most important implication of these findings is that nearly half of the study population have antibodies detected on MAT to the non-pathogenic serovar Patoc, and this proportion is reduced to around a third of the population when pathogenic serovars are tested for. MAT is often used for acute diagnosis of leptospirosis; testing for leptospirosis using MAT based on non-pathogenic strains may result in false positives in up to 20% of patients. It is also noteworthy that 25% of individuals who tested negative for saprophytic serovars were positive for pathogenic serovar MAT; this could result in an unacceptable number of false negatives with the use of saprophytic MAT. There is little doubt that testing for serovar specific MAT is essential, although this is more cumbersome and time consuming, as testing has to take place against an ever-growing panel. Identification of serovars prevalent in the locality tested will play an important role in fine-tuning this test.

One of the key problems in the use of MAT is that the test cannot differentiate between IgM and IgG antibodies in the serum of the sampled individual. Thus, for MAT to be useful to the clinician managing a patient with a clinical syndrome of leptospirosis, the MAT titre cut-off for diagnosis of acute infection must be based on the relevant baseline MAT titres in the healthy community and locality to which the patient belongs. If this factor is not taken into consideration, it can result in a high number of incorrect diagnoses. This is of particular relevance in settings where many other tropical infectious diseases, such as dengue, hantavirus infection, typhus and viral hepatitis, are common. The use of a single MAT titre cut-off should be reconsidered in the light of these findings. Determination of region and serovar specific MAT cut-offs should be performed in different settings, if they are to be relevant and accurate. The findings of this study indicate that a diagnostic cutoff of ≥1/320 or ≥1/400, as currently often advocated, would not be appropriate for most serovars in this study population.

### Lack of seroprevalence data from Sri Lanka

Prior evidence on seroprevalence in Sri Lanka originates from studies in the 1960s to 1970s. The first of these studies included a high-risk population along the Colombo -Negombo- Puttalam canal and high-risk groups selected from the farming population, and reports a leptospiral antibody seroprevalence of 23.8%[20]. This data was subsequently reanalyzed using a panel of serovars which indicated a seroprevalence of leptospirosis of 17.7%. among 51 serum samples from healthy individuals. Another study on seroprevalence of *Leptospira* antibodies based on occupational exposures using the macroscopic agglutination test was conducted in 1967 [21]. This study showed seropositivity (titer ≥1/30) in a range of occupational groups where sugar cane farmers had the highest seroprevalence at 72.7%. The current study, which is the only recent study on community based seroprevalence based on MAT in Sri Lanka, fills a significant void in the understanding of the disease.

### Pyrogenes was the most commonly detected serovar

The serovar Pyrogenes was the most commonly detected serovar in this community-based study. More than 20 serovars have been documented from Sri Lanka from four species, in serological studies; *L*. *interrogans*, *L*. *kirschneri*, *L*. *borgpetersenii* and *L*. *santarosai*. Further advanced techniques such as multi- locus sequencing based genotyping shows that at least 13 genotypes are causative agents of leptospirosis in Sri Lanka, which includes several *Leptospira* spp. serovar Pyrogenes [22] We also noted regional variation in common serotypes in some analyzed clusters, such as Bankinang, Ratnapura and Australis. This might be due to unique ecological, reservoir, and transmission characteristics, and requires further evaluation.

### Associations and regression models

The traditional risk exposures such as occupational exposure including farmers and others engaged in agriculture, contact with stagnant water, bathing and swimming in local rivers and lakes were associated with a positive pathogenic MAT. The significant associations with male gender are probably because most high-risk occupations and exposures are traditionally associated with men in these communities. The fact that seroprevalence was higher in low income settings may relate to environmental factors as well as knowledge, attitude and practice determinants. Interestingly, exposure to floods and the presence of rats in the vicinity of households had negative associations with pathogenic serovar MAT. The negative associations of rats in the vicinity on seroprevalence for pathogenic MAT as well as the relatively higher seroprevalence in those exposed to cattle and buffaloes is important to elucidate further. This may be due to poor reliability of reporting of respondents or due to the emergence of other animal reservoirs for the disease. Previous studies of samples obtained from cattle kidneys in Colombo reported significant positivity for *Leptospira* (20.2%) using PCR techniques[23]. These findings are consistent with another study identifying PCR positivity in urine samples obtained from bovine animals[24].

Seroprevalence was lower in patients who reported exposure to floods, which is usually reported as a risk factor of the disease. A careful characterization of this exposure is required to investigate this further. Seroprevalence was higher in proximity to water sources within buffer zones of 250m and 100m. A clear clustering effect was seen for certain serovars, as demonstrated by the GIS maps. This may be due to unique transmission and ecological characteristics of the serovar. Further studies should explore these associations in more detail.

The findings of this study add to the existing body of evidence based on seroprevalence studies in other endemic and non-endemic regions. Studies from Malaysia among wet-market workers demonstrated a seroprevalence of 33.6% (95% CI  =  27.5, 39.7) and predominance of the serovar Autumnalis[15]. Other studies have shown high seroprevalence among town service workers, palm oil planters [25], market workers and food handlers[14]. Studies from Pakistan demonstrate environmental variation in seroprevalence, with the highest seroprevalence noted in the sub-tropical climatic regions [26]. Seroprevalence data from the Andaman Islands demonstrate high prevalence among agricultural communities[27]. Other seroprevalence studies are reported from the Pacific Islands [28], Trinidad, Barbados [29], Puerto Rico [18] and Mexico [17] among others, each with varying seroprevalence and unique region and locality specific risk factors.

### Limitations and recommendations for future studies

This study used MAT cutoffs of 1/40 for determination of seroprevalence. The cut-off value of MAT usually depends on the baseline in the community in a geographical area and varies from laboratory to laboratory, with most studies using cutoffs of 1/100 [16, 25]. However, there is evidence for lower cut-offs for sero-surveys in asymptomatic high-risk groups such as those evaluated in our study [30].

This study used a panel of 11 pathogenic serovars for MAT testing. It is possible that certain important serovars present in the community might have been missed as a result. However, this panel undergoes expansion and revision with time, based on epidemiological trends. A recent hospital-based analysis explored the emergence of the serogroup Tarrasovi in patient samples [31]. It is therefore pertinent that the serum samples from this study be preserved and repeatedly analyzed using a further expanded serovar panel.

Another limitation is that the serovars tested against could be mixtures of different species or serogroups, and thus the antigen-antibody compatibility may not be as precise as it would be if the identical species-serovar was tested for. This may have resulted in the community titres for some serovars being identified as falsely low. A bigger study with more detailed identification of serovars, using species specific serovars, would be required to overcome this limitation.

This study was based on a cross-sectional design and therefore has inherent limitations of causality with regards to many of the associations presented above. Prospective modelling studies could address these associations further.

### Conclusions

This study provides valuable data on the seroprevalence and associations of leptospirosis in the district of Colombo in Sri Lanka, and fills a significant void in community-based studies originating from the region. The associations with risk factors and geographic features should be utilized for design of policy and prevention strategies. Future studies should also further explore how disease transmission to humans is influenced by the complex interactions between humans, animals, and the environment. Further studies should also focus on mathematical modelling with weather patterns and determinants of health economics. A revisit of diagnostic thresholds for MAT titres is also warranted.
